# Impact of extending direct antiviral agents (DAA) availability in France: an observational cohort study (2015-2019) of data from French administrative healthcare databases (SNDS).

**DOI:** 10.1016/j.lanepe.2021.100281

**Published:** 2021-12-11

**Authors:** Stanislas Pol, Fayssoil Fouad, Magali Lemaitre, Ingrid Rodriguez, Olivier Lada, Pascaline Rabiega, Elias Benabadji, Françoise Roudot-Thoraval

**Affiliations:** a Université de Paris, département d'hépatologie et d'addictologie, 75014 Cochin (AP-HP), Paris, France; b IQVIA, 92400 Courbevoie, France; c Gilead Sciences, 92100 Boulogne Billancourt, France; d Service d'hépatologie, Henri-Mondor (AP-HP), 94010, Créteil, France

## Abstract

**Background:**

Direct antiviral agents (DAAs) became available in France in 2014 for the treatment of chronic hepatitis C (CHC) in patients with severe fibrosis (prioritized access); in 2017, DAAs became available to all CHC patients (universal access). We evaluated the impact of extending DAA availability on CHC patient care, especially on screening and time to treatment.

**Methods:**

Adult patients affiliated with the national health insurance system (SNDS) who were screened or treated for CHC between 2015 and 2019 were included. Algorithms were developed to identify at-risk subpopulations.

**Findings:**

The proportion of screened patients increased by 1% between 2015 and 2019, from 4·6**%** to 5·6%. The main nonexclusive risk factors for CHC were psychiatric conditions (27%), drug use (21%) and HIV positivity (11%); more than 50% of psychiatric patients had additional risk factors, mainly drug use with a 38% to 52% overlap.

The median interval between the last screening test and treatment initiation decreased from 64 days in 2015 to 37 days in 2019.

During the study period, 71,466 patients began CHC treatment (median age 55 [48-62]; 59% male), including 46% of “at-risk” patients with an increase in treatment initiation by 44% between 2015 and 2017 and a decrease of 46% between 2017 and 2019. Only 2,212 (3%) patients were treated at least twice.

Among treated patients, the proportion of HIV+ patients decreased from 19% to 8% (prioritization consequence), while the proportions increased in the other at-risk subpopulations.

**Interpretation:**

we showed that policies extending DAA availability are associated with a screening increase and a decrease in the time to treatment initiation, while universal access led to a surge in treatment initiations in 2017. This study may also contribute to improving the cascade of care in the at-risk subpopulations. For instance, by pointing out their relative importance, especially for the psychiatric subpopulation, it highlights the importance to address them with tailored policies.

**Research in context**

**Evidence before this study**

The at-risk subpopulations (migrants, inmates, HIV+ patients, psychiatric patients and drug users) were chosen in accordance with the existing literature about HCV risk factors.

Our study is the first epidemiological study on HCV patients treated with DAAs in France. There were no existing studies that provided information about treated patients since the introduction of DAAs or that assessed the impact of the evolution of national public policy regarding DAA access on CHC patient care.

**Added value of this study**

Evaluate the impact of policies extending DAA access on CHC patient care: screening, treatment initiation, and time between screening and treatment initiation.

Describe evolution of epidemiological data on patients treated for CHC, and especially assess the proportion of at-risk subpopulation among treated patients with DAAs.

Assess the results of the public health policy that aimed at prioritizing coinfected HIV+ patients for screening and treatment initiation.

Better characterization of at-risk subpopulations, their relative weight and their overlap to improve the cascade of care in these subpopulations.

**Implications of all the available evidence**

Psychiatric patients represent the most important subpopulation at risk among treated patients. Higher screening efforts targeting psychiatric patients are therefore needed to achieve elimination in France. As the psychiatric population represents a large group (27% of treated patients), further studies are needed to better characterize this subpopulation and identify potential subgroups at risk.

Alt-text: Unlabelled box

## Introduction

Chronic hepatitis C virus (HCV) affects over 71 million individuals worldwide.^1^ In France, it was estimated in 2011 that approximately 192 700 individuals were concerned.^2^ CHC is a major cause of liver cancer and is associated with significant fibrosis or cirrhosis, depending on the presence or absence of aggravating factors.^3^ Chronic hepatitis C (CHC) is also associated with extrahepatic manifestations, such as cryoglobulinemic vasculitis and chronic inflammation.^4^

The arrival of new direct-acting antivirals (DAAs) in 2014 substantially modified the management of hepatitis C virus infection with higher cure rates (over 95% for all major genotypes), good tolerance and short-course therapy (8-24 weeks).^5^^,^^6^ Second-generation pangenotypic DAAs, a major medical breakthrough, significantly reduced HCV-related morbidity and mortality.^7^

Given the results associated with second-generation DAAs, the World Health Organization (WHO) has set the goal of eliminating HCV infection by 2030. Accordingly, the French National Public Health Program 2018-2022 established objectives for the elimination of hepatitis C by 2025 in France, with 120,000 people treated with DAAs by the end of 2022.

In France, following the recommendations of the Haute Autorité de Santé (HAS),^8^ a prioritized access policy to DAAs was initially enforced. From November 2014 to June 2016, access to DAAs was initially restricted to patients with severe liver disease (cirrhosis) or extrahepatic disease (HIV, cryoglobulinemia and B-cell lymphoma),^9^ The prioritized access was then extended in June 2016 to patients with stage F2 fibrosis and patients with a high risk of transmission (inmates, drug users and pregnant women).^10^ Additionally, during the prioritized access period, DAAs could only be prescribed in hospital settings after a multidisciplinary concertation. Eventually, in April 2017, following the evolution of the HAS recommendations,^11^ a universal access policy replaced the prioritized access policy: DAAs became available for all CHC patients, and prescriptions were made possible outside of the hospital by specialists in ambulatory care. Consecutively, in March 2018, DAAs were offered in all retail pharmacies, and in May 2019, every physician was granted the ability to prescribe pangenotypic second-generation DAAs.

We hypothesized that the evolution of DAA access policies in France had an impact in CHC patients care management: we put in perspective the gradual expansion of DAA availability with evolutions of the screened population, treatment initiations and time between screening and treatment initiation between 2015 and 2019. This analysis was conducted in the general population and in subpopulations considered to be at high risk of HCV infection, namely drug users, migrants, inmates and Human immune deficiency virus (HIV)-infected populations.^12^^,^^13^

## Methods

### Data source

This observational cohort study was based on data from the French administrative healthcare databases (SNDS).^14^ Several databases are included in the SNDS. For this study, the DCIR *(Données de Consommation Inter-régime)* and the PMSI (*Programme de Médicalisation des Systemes d'Information*) were used, taking advantage of database linkages through unique anonymous identifiers. The DCIR covers approximately 95% of the French population, with different schemes based on employment status. Each individual contains information about outpatient medical care and sociodemographic characteristics, as well as medical information about the presence of chronic diseases included in a list of eligible chronic conditions and the patient's status with respect to full reimbursement of care for long-term diseases (LTD) listed, encoded by codes International Statistics Classification of Diseases and Related Health Problems, Tenth Revision (ICD-10) codes. The PMSI encompasses individual medical information about all hospital admissions in France, with discharge diagnoses (encoded by ICD-10 codes) and medical procedures.

### Study population

#### Individuals screened for HCV

The HCV screening population comprised all adult individuals with at least one reimbursement for enzyme immunoassay (EIA) serodiagnosis to detect anti-HCV antibodies or for quantitative HCV RNA detection, tests performed in ambulatory care settings, private hospitals or private consults within public hospitals.

### Patients with treatment initiation

All patients aged over 18 years treated for CHC (interferons or DAAs) from January 1^st^, 2015, to December 31^st^, 2019, and with no history of CHC treatment from January 1^st^, 2014, to the enrolment date, were selected. In case of treatment by interferons, LTD and/or hospitalization for CHC were required. Treatments considered for inclusion were interferons or DAAs, as interferons were still prescribed for the treatment of CHC in 2015.

Among patients with treatment initiation, we analysed 2 subgroups: *1. the “at-risk subpopulation”* and *2. “retreated patients”,* i.e., those treated at least twice*.*

The “at-risk population” comprised five subgroups (migrants, prisoners, HIV positive patients, psychiatric patients and drug users) identified through algorithms described in the supplementary Table S1. We are working on medico-administrative data and there is no data collected regarding active or inactive drug use. If the notion of drug injection is not captured directly the active drug users are often populations with psychiatric disorders that we therefore capture elsewhere. At the opposite, the opiate substitution therapy (OST) is captured and we know that a significant proportion of “inactive” drug users under OST still perform injections.

The retreated subgroup comprises patients with viral failure or reinfection; these patients were identified by the presence of 2 treatment courses for CHC, with an interval of more than 3 months between courses, without information about the cause of retreatment, treatment failure including nonadherence or relapse, or reinfection.

### Statistical approach

The results are presented as medians [interquartile ranges] for continuous variables and numbers or percentages for categorical variables.

The Mann-Kendall (MK) test was used to statistically assess whether there was a continuous upward or downward trend among the number of patients over time (significance level set at 5%).

The duration of DAA treatment was described for the entire treated population and for at-risk subpopulations.

### Role of the funding source

The study sponsors (i.e., the authors affiliated with Gilead Sciences) initiated the study by solicitating IQVIA France for accessing the SNDS databases, performing analysis on raw data (data collection) and writing the study report. According to French laws on medical data privacy, IQVIA proceeded to the SNDS database access process requiring ethics approval, including approval from CESREES (*Comité d'Expertise pour les Recherches, les Etudes et les Evaluations dans le domaine de la Santé*) and CNIL (*Commission Nationale de l'Informatique et des Libertés)*. Data access was delivered by the CNAM (*Caisse nationale d'assurance maladie*) after signing an agreement.

The study sponsors also solicitated the expertise of Pr. Stanislas Pol and Dr. Françoise Roudot-Thoraval to interpret the set of data and recommend, if relevant, further analysis of the raw data. Both Pr Pol and Dr Roudot-Thoraval received grants from Gilead Sciences.

Along with the other authors, the authors affiliated with Gilead Sciences participated in the analysis and interpretation of data and the decision to submit the paper for publication.

## Results

### Individuals screened for HCV

Between 2015 and 2019, the population screened for HCV in France increased from 2.9 million to 3.7 million. Indeed, the rate of the screened population reached 5·63% of French inhabitants in 2019 versus 4·38 % in 2015 (+1·25%). Nearly two-thirds of the screened population was women, with a median age of 36 years [28-51]. No changes in these characteristics were observed between 2015 and 2019 (Table 1). More than 80% of screened women were less than 55 years old, compared to 70% of screened men.

**Table 1:** Hepatitis C screening in the general population per year, 2015-2019.

	Year	2015	2016	2017	2018	2019
**Number of screened patients**	**N**	2,908,829	3,069,080	3,235,197	3,486,815	3,782,398
**Proportion of screened patients in the French population**	**%**	4.38	4.61	4.84	5.20	5.63
**Characteristics of screened patients**						
**Female**	**N (%)**	1,805,634 (62.7)	1,895,381 (62.3)	1,992,918 (62.0)	2,142,747 (61.8)	2,316,154 (61.6)
**Sex Missing Data**	**N**	29,826	26,312	22,418	19,475	20,378
**Age, years**	**Median (Q1-Q3)**	36.0 [28.0 - 51.0]	36.0 [28.0 - 51.0]	36.0 [28.0 - 51.0]	35.0 [27.0 - 51.0]	35.0 [27.0 - 50.0]

In the general population among 59,779 patients with HCV screening tests and treatment initiation between 2015 and 2019, the median time between the last test performed and the initiation of treatment decreased from 77 days in 2016 (more than 2 months) to 37 days in 2019. Notably, this interval was 64 days in 2015. This interval even reaches 20 days in HIV+ patients, highlighting the rapid management of these patients in particular (Table 2).

**Table 2:** Time to treatment initiation after hepatitis C screening in the general population and subgroups per year, 2015-2019.

	Year	2015	2016	2017	2018	2019
**Number of patients with incident-initiated treatment and HCV screening**	**N**	12,517	13,789	15,896	10,781	6,796
Interval*between test and initiation of treatment, days	**Median**	64.0	77.0	60.0	53.0	37.0
	**Q1 - Q3**	[8.0 -171.0]	[24.0 - 168.0]	[20.0 - 123.0]	[21.0 - 107.0]	[16.0 - 75.0]
**Number of psychiatric patients with incident-initiated treatment and HCV screening**	**N**	3,326	3,641	3,972	2,811	1,722
Interval*between test and initiation of treatment, days	**Median**	70.0	84.0	62.0	55.0	38.0
	**Q1 - Q3**	[8.0 -196.0]	[25.0 -187.0]	[21.0 -131.0]	[21.0 -115.0]	[16.0 -78.0]
**Number of HIV-patients with incident-initiated treatment and HCV screening**	**N**	2,158	1,728	1,019	763	542
Interval*between test and initiation of treatment, days	**Median**	57.0	43.5	51.0	38.0	20.0
	**Q1 - Q3**	[7.0 -168.0]	[5.0 -120.0]	[11.0 -122.0]	[10.0 -95.0]	[6.0 -50.0]
**Number of DU with incident-initiated treatment and HCV screening**	**N**	2,263	2,726	2,997	2,333	1,548
Interval*between test and initiation of treatment, days	**Median**	86.0	88.0	70.0	60.0	40.0
	**Q1 - Q3**	[20.0 -244.0]	[31.0 -199.0]	[27.0 -146.0]	[22.0 -124.0]	[19.0 -83.0]

⁎ The interval was calculated using the last test performed before the initiation of treatment during the study period.

### Patients with treatment initiation

**Figure 1:**
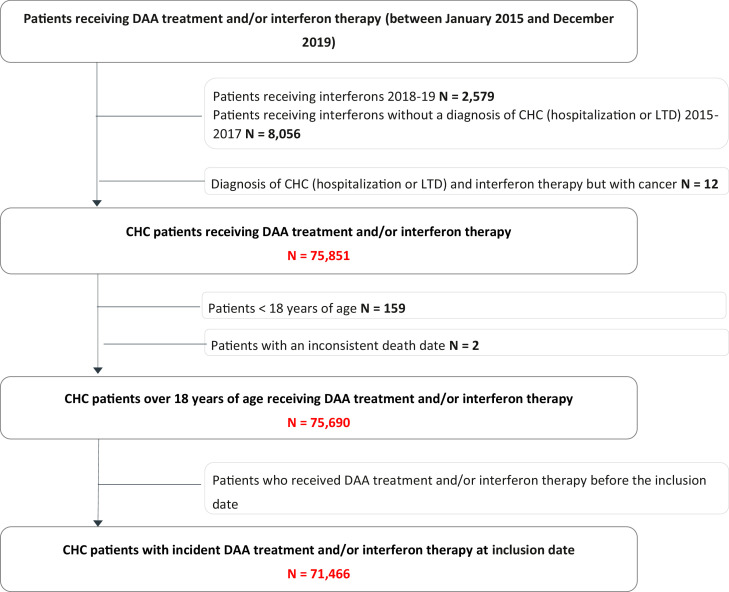
Flowchart.

A total of 71,466 patients with treatment initiation (DAAs: 99·8%) between 2015 and 2019 were identified (Figure 1). Overall, between 2015 and 2019, patients were mostly male (58·7%), and the median age was 55·0 years [48·0-62·0]. The proportion of men in the treated population decreased from 63·7% in 2015 to 54·2% in 2017 and then increased to 60·7% in 2019. During the study period, more than half of the patients (50·2%) were 55 years of age or older. This proportion declined throughout the study period (from 56·1% in 2015 to 48·0% in 2019). More than 48% of the screened patients were under 35 years old, while only 6% of the treated patients were in that age class.

**Figure 2:**
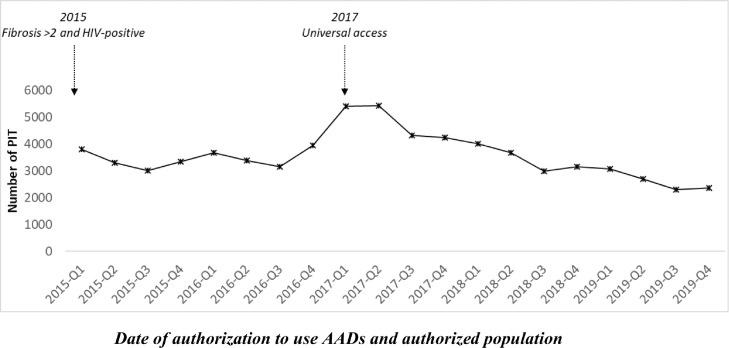
Quarterly number of patients initiating treatment (PIT), 2015-2019.

As shown in Figure 2, between 2015 and 2017, the number of patients initiating treatment increased by 44% (from 13,476 to 19,459). Overall, a monthly significant increasing trend (p-value 0·0102) was observed. Between 2017 and 2019, a monthly significant decreasing trend was observed, with an overall decrease of 46·2% (from 19,459 to 10,477) (p-value <0·001).

The duration of DAA treatment decreased from a median of 84 days [79-89] in the first quarter of 2015 to a median of 73 days [53-83] in the third quarter of 2019 (Table S2).

### At-risk subpopulations

Overall, more than half of the treated patients (54%) were not at risk. During the study period, the proportion of non-at-risk patients slightly varied from 53% in 2015 to 58% in 2017 and finally returned to the initial rate of 52% in 2019. Over the entire study period (2015 to 2019), nonexclusive “at-risk” factors were psychiatric conditions (27%), drug use (21%), HIV positivity (11%), migrant status (5%), and imprisonment (4%).

**Figure 3:**
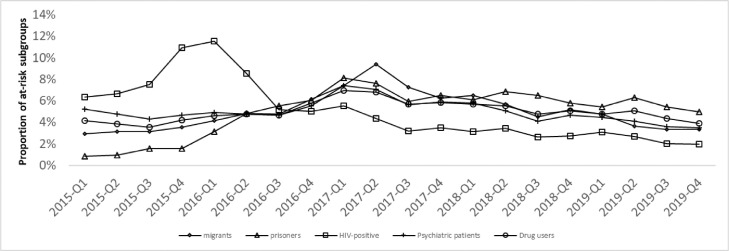
Time trend of the proportions of high-risk subgroups among treated patients, 2015-2019.

Sixty-two percent of the HIV+ patients, targeted by the prioritized access policy, were treated during the first two years of the 5-year study period (2015-2016). In comparison, only 39% of the treated psychiatric patients, 36% of the treated DUs, 33% of the treated migrants and 24% of the treated inmates initiated treatment during the same period (2015-2016). All subpopulations other than HIV+ patients reached a patient treatment proportion of 50% in 2017, one year after the HIV patients. The maximum proportion of treated patients in one quarter was reached in Q1 2016 for HIV+ patients (12%), Q1 2017 for psychiatric patients (7%), Q1 2017 for DUs (7%), Q2 2017 for migrants (9%), and Q1 2017 for prisoners (8%) (Figure 3).

The rate of HIV+ patients among all the treated patients decreased substantially from 26% at the end of 2015 to 8% in Q1 2017 and remained stable until Q4 2019. The DU subpopulation among the treated patients increased during the study period, from 16% to 24%. The inmate subpopulation among treated patients increased from 1% to 6% at the beginning of the study period (2015-2016) before remaining stable at 6% until Q4 2019. The proportions of the two remaining subpopulations remained stable between 2015 and 2019. The proportion of treated psychiatric patients was approximately 25%, while the proportion of treated migrants was approximately 5% (Figure S1).

The subpopulation of treated psychiatric patients increased by 37% between 2015 and 2017, from 3,628 patients to 4,958, and decreased by 39.5% between 2017 and 2019, from 4,958 to 3,000. The median age was 53 years old during the study period. The proportion of men decreased from 66% in 2015 to 59% in 2017 and returned to the initial rate in 2019 (Table S3).

More than half of psychiatric patients (51% to 57% depending on the year) had additional risk factors, mainly drug addiction (alone or with other risk factors), with the overlap ranging from 38% to 52% depending on the year. It should be noted that 58%-60% of the DU subpopulation also had psychiatric conditions depending on the year, showing a strong association between these two at-risk subpopulations.

Moreover, the profile of the psychiatric subpopulation evolved over the study period. The proportion of psychiatric patients with only drug addiction (as another risk factor) gradually increased from 25% in 2015 to 39% in 2019. In parallel, among the psychiatric subpopulation, a decline in the proportion of HIV+ patients with or without drug addiction was observed. Conversely, the proportion of psychiatric patients with other combinations of cofactors increased from 3% to 11% (Figure S2).

### Retreated subpopulation (population treated at least twice)

A total of 2,212 (3·1%) patients were retreated (namely, treated at least twice). The SNDS databases do not provide information about whether retreatment was due to non-adherence, virologic failure or reinfection. The median age was 55 years [48-62], and 70% of retreated patients were men (Table S4). Most of the retreated patients identified during the study period were retreated in 2018 (875 patients; 40%). In 48·4% of cases, an interval greater than 12 months between the first and second courses was observed. Interestingly, 31·8% of patients were retreated between 6 and 12 months after the first course.

More than half of the patients in the retreated subpopulation (54·9%) were identified to be at-risk. DUs and psychiatric patients accounted for 21·8% and 28·7% of this population, respectively. These distributions were in accordance with their proportions in the population with first treatment initiation. In contrast, HIV+ patients accounted for 23·2% of the patients treated twice versus 11% in the population with first treatment initiation.

### HCV-drug prescribers

HCV drug prescriptions were mainly administered by hospital-based hepato-gastroenterologists during the entire study period. However, the proportion of prescriptions administered by private practitioners increased from 5% in 2015 to 20% in 2019, illustrating the ramp up of ambulatory care. The prescription authorization of two pangenotypic DAAs for all physicians on May 20, 2019, also had an impact on the prescriber profile. Indeed, the proportion of prescriptions administered by general practitioners (GPs) averaged 4% in 2019, but it should be noted that it was implemented only during the last 6 months of 2019.

## Discussion

The shift, from prioritized access in hospital settings only to universal access in both hospital and ambulatory care settings, correlates with a surge in treatment initiations between 2016 and 2017 (+37%). These data suggest the beneficial effects of extending DAA access to achieve the 2022 target of 120,000 patients treated: by the end of 2019, 71,466 patients had initiated therapy. Moreover, the fact that treatment initiations have been declining since 2018 (- 46% between 2017 and 2019) could suggest that the reservoir of patients is shrinking as fewer patients are diagnosed, while we showed that screening efforts have continued to increase.

This increase in the screened population must also be related to the extension of DAA access. The mobilization of ambulatory care resources, along with the possibility of treating younger outpatients with a less advanced form of the disease, certainly contributed to ramp up screening efforts and to ensure the success of the multiple hepatitis C screening campaigns launched.^15^ In that regard, among treated patients, the proportion of non-at-risk patients rose from 53% to 58% between 2015 and 2017, while the absolute number of patients treated increased, highlighting that the change in access policy accelerated care among all HCV patients. The resurgence in the proportion of females, from 36.3% in 2015 to 39·3% in 2019, could also be associated with the extension of treatment to those with less severe hepatitis C (progression rate of fibrosis is slower in women than in men, and the risk of hepatocellular carcinoma is lower, probably due to different exposures to risk factors^16^ such as alcohol consumption and hormonal protection).^17^

Another notable element is the steady reduction of the time period between screening and initiation of treatment from 2015 to 2019. This time period went from a median of more than two months to slightly more than one month, indicating an improvement in patient management following the progressive extension of DAA access.

Additionally, this study is the first to use the SNDS database to estimate and describe almost the entirety of CHC patients treated with DAAs in France from 2015 to 2019. It therefore provides an exhaustive overview of the patients’ characteristics. In particular, the development of several algorithms allowed us to identify at-risk populations and highlight their specific features in terms of care management and access to care.

Interestingly, having a psychiatric condition appears to be the most common risk factor: psychiatric patients accounted for 27% of treated patients and 21% of DUs, with an important overlap between these two groups (38% to 52% depending on the year).

Regarding other at-risk subpopulations, interestingly, the prioritized access policy for HIV+ patients (11%) proved to be effective, as this subpopulation had the highest treatment rate at the beginning of the study period (2015-2016), while the number of treatment initiations decreased significantly by the end of the study period. In comparison, the psychiatric and DU subpopulations followed the same trend as the general population, with a peak in 2017 followed by a decrease in 2018, which might indicate an earlier shrinkage of the patient reservoir in the HIV+ subpopulation.

The specific dynamics of treatment initiation among inmates should also be mentioned. It differs significantly from the general population, with a sharp increase in treatment initiations by the end of 2015 and an acceleration in 2016. The specificity of this dynamic can be linked to the evolution of the rules regarding DAA access in prison. Before May 2015, only hospitalized inmates had access to DAAs: a derogatory access scheme was then enforced to grant access to non-hospitalized inmates.^18^ Moreover, CHC inmates were granted universal access earlier than the general population in June 2016 due to higher risks of transmission in prison.

The retreated population was also of interest due to the difference in characteristics between the populations, emphasizing the need for further studies to differentiate non-adherence, virologic relapse and reinfection. The proportion of women was lower (29·6% vs 41·3%) in the retreated population, possibly because of differences in risk behaviours – alcohol consumption, drug use and reinfection – in men who have sex with men (MSM). The non-at-risk population accounted for 45·1% of the retreated population, which highlights that the risk of retreatment is prevalent in any profile of patients. However, HIV+ patients accounted for 23·2% of patients retreated twice versus 11% in the treated population, consistent with the higher risk of HCV reinfection in cases of HIV coinfection, especially in MSM.^19^

Eventually, as this study provides an acute understanding of HCV treatment management and its evolution since 2015, it also highlights the remaining challenges to reach HCV elimination in France by 2025. Especially in the COVID-19 pandemic context, likely to have negatively impacted CHC care, all levers should be used to improve collective mobilization for elimination.

First, data show that the increased screening activity failed to accurately target infected populations, as there was a noticeable difference in the demographic profile between the screened population and the treated population. There were more females than males among the screened patients and more males than females among the treated patients. Notably, a median age of 36 years [28-51] was observed in the screened population versus a median age of 55 years [48-62] in the treated population. This result may be explained by screening in women of childbearing age. However, since the *Haute Autorité de Santé* (HAS) has advised against universal screening,^20^ targeted screening campaigns targeting psychiatric patients 50 years and older appear necessary to enhance the efficiency of screening efforts in France.

Second, while patients with psychiatric conditions account for more than one out of four CHC patients, treatment initiation needs to be carefully monitored for these patients^21^ due to high rates of medication non-adherence^22^^,^^23^ and high risk of drug interactions in the psychiatric population. If the release of DAAs provides a great opportunity to treat patients with psychiatric conditions,^24^ access to DAAs for inpatients often remains a challenge.^25^ As treating CHC psychiatric patients requires a tailored approach and careful monitoring, hospital stay should instead be a key opportunity to effectively and safely treat these patients. Additionally, more studies are needed to better characterize this large subpopulation since defining psychiatric condition as a risk factor constitutes a broad spectrum that can potentially be refined.

Third, if some GPs seized the possibility of prescribing pangenotypic DAAs opened in May 2019, the proportion of prescriptions made by GPs remained too low (4%) to have a significant impact.

Last, this study presents some limitations inherent to the databases. The algorithms implemented partly for the first time in SNDS to identify HIV+ patients, DUs and psychiatric patients were mostly based on the occurrence of hospitalization, and outpatient care data included only the drug regimen since the diagnosis during consultation was not available in the database. Especially DUs can be underestimated because of the complexity of their management and the fact that they were mainly captured by the ICD-10 codes of hospitalization and/or the use of opiate substitution therapy. Moreover, follow-up of the migrant subpopulation represented a challenge. The information about screening tests extracted from the databases was limited to the tests executed in ambulatory care, private hospitals and external clinics of public hospitals. Consequently, patients who receive a test in public inpatient care could have a shortened duration between screening and initiation of treatment because they are already under care and will likely undergo a consultation. Importantly, test results were not available in the database, so profile descriptions of screened patients, not patients with a positive test result, were realized.

Finally, clear limitation of our study is our inability to clearly identify people who inject drugs: in most Western countries that are aiming to eliminate hepatitis C as a public health threat, this is the population that remains hardest to engage, and also has the highest risk of transmission. This key population (in prisons, in health care units devoted to drug users) has been partially treated in our results and we cannot ascertain the actual proportion. This population requires dedicated models of care that reduce barriers to the cascade of care, which include harm reductions policies, easy access to therapy and follow-up  for the detection of reinfection and its treatment which are well-implemented in France. We are proceeding to a specific analysis of the psychiatric population in order to provide additional elements to this study but it will not be possible to address the subpopulation of the active drug users.

## Conclusion

This study highlights the impact of the change in policy regarding DAA access on CHC patient care management. Along with the opening of ambulatory care prescription and dispensation, the shift from prioritized access to universal access is associated with a significant increase in treatment initiations in 2016 and 2017, higher screening efforts and a reduction in the interval between screening and treatment initiation.

As a result, France now seems on the right track to achieve elimination by 2025. However, the data also suggest that fully achieving elimination would require better targeted screening efforts, notably on psychiatric patients and elderly individuals over 50 years. Indeed, by improving the characterization of at-risk subpopulations, this study revealed the importance of their relative weight and the capital need to address them with tailored policies to avoid the persistence of micro-reservoirs.

## Declaration of Competing Interests

Stanislas Pol has received consulting and lecturing fees from Janssen, Gilead, MSD, Abbvie, Biotest, Shinogui, Viiv, LFB and grants from Bristol-Myers Squibb, Gilead, Roche and MSD.

Françoise Roudot-Thoraval has received consulting and lecturing fees from Gilead and AbbVie.

Magali Lemaitre: Employee at IQVIA, commissioned by Gilead Sciences to access the SNDS database and perform the analysis.

Fayssoil Fouad: employee at IQVIA, commissioned by Gilead Sciences to access the SNDS database and perform the analysis.

Ingrid Rodriguez: employee at Gilead Sciences

Olivier Lada: employee at Gilead Sciences

Pascaline Rabiega: employee at IQVIA, commissioned by Gilead Sciences to access the SNDS database and perform the analysis

Elias Benabadji: employee at Gilead Sciences

## Appendix Supplementary materials

Image, application 1

Image, application 2

Image, application 3

Image, application 4

Image, application 5

Image, application 6

## References

[1] WHO. Hepatitis C. http://www.who.int/mediacentre/factsheets/fs164/fr/ (accessed Feb 17, 2020 ).

[2] Pioche C, Pelat C, Larsen C (2016). Estimation de la prévalence de l'hépatite C en population générale, France métropolitaine, 2011. Bull Epidemiol Hebd.

[3] Hoofnagle JH. (2002). Course and outcome of hepatitis C. Hepatology.

[4] Pol S, Vallet-Pichard A, Hermine O (2018). Extrahepatic cancers and chronic HCV infection. Nat Rev Gastroenterol Hepatol.

[5] Bryan-Marrugo OL, Ramos-Jiménez J, Barrera-Saldaña H, Rojas-Martínez A, Vidaltamayo R, Rivas-Estilla AM. (2015). History and progress of antiviral drugs: From acyclovir to direct-acting antiviral agents (DAAs) for Hepatitis C. Med Univ.

[6] Collins LF, Chan A, Zheng J (2018). Direct-acting antivirals improve access to care and cure for patients with HIV and chronic HCV infection. Open Forum Infect Dis.

[7] Carrat F, Fontaine H, Dorival C (2019). Clinical outcomes in patients with chronic hepatitis C after direct-acting antiviral treatment: a prospective cohort study. Lancet.

[8] Haute Autorité de Santé. Recommandation du Collège de la HAS, prise en charge de l'hépatite C par les médicaments anti-viraux à action directe (AAD). Jul, 2014. https://www.has-sante.fr/upload/docs/application/pdf/201407/hepatite_c_prise_en_charge_anti_viraux_aad.pdf (accessed Apr 28, 2021).

[9] Journal Officiel de la République Française. Arrêté du 18 novembre 2014 relatif aux conditions de prise en charge de spécialités pharmaceutiques disposant d'une autorisation de mise sur le marché inscrites sur la liste visée à l'article L. 5126-4 du code de la santé publique. NOR: AFSS1426759A ELI. 18 novembre 2014. https://www.legifrance.gouv.fr/eli/arrete/2014/11/18/AFSS1426759A/jo/texte (accessed Apr 28, 2021)

[10] Journal Officiel de la République Française. Arrêté du 10 juin 2016 relatif aux conditions de prise en charge de spécialités pharmaceutiques disposant d'une autorisation de mise sur le marché inscrites sur la liste visée à l'article L. 5126-4 du code de la santé publique. NOR: AFSS1613575A ELI. https://www.legifrance.gouv.fr/eli/arrete/2016/6/10/AFSS1613575A/jo/texte. (accessed Apr 28, 2021)

[11] Haute Autorité de Santé. Recommandation du Collège de la HAS, pris en charge de l'hépatite C par les médicaments antiviraux d'action directe (AA), élargissement du périmètre de remboursemnet. https://www.has-sante.fr/portail/upload/docs/application/pdf/201612/recommandation_college_hepatite_c.pdf (accessed Apr 28, 2021 ).

[12] European Association for the Study of the Liver (2020). (EASL) recommendations on treatment of hepatitis C: final update of the series. J Hepatol.

[13] AASLD-IDSA. Recommendations for testing, managing, and treating hepatitis C. 2017. http://www.hcvguidelines.org (accessed Jul 16, 2017).

[14] Tuppin P, Rudant J, Constantinou P (2017). Value of a national administrative database to guide public decisions: from the système national d'information interrégimes de l'Assurance Maladie (SNIIRAM) to the système national des données de santé (SNDS) in France. Rev Epidemiol Sante Publique.

[15] SOS Hépatite. Campagne - savoir c'est guérir: savoir c'est guérir. https://www.savoir-c-guerir.com/ (accessed Feb 17, 2020).

[16] Rosenberg SD, Goodman LA, Osher FC (2001). Prevalence of HIV, hepatitis B, and hepatitis C in people with severe mental illness. Am J Public Health.

[17] Di Martino V, Lebray P, Myers RP (2004). Progression of liver fibrosis in women infected with hepatitis C: long-term benefit of estrogen exposure. Hepatology.

[18] Légifrance. Circulaire N° DGOS/R1/R4/DSS/1A/1C/2A/2015/148 du 29 avril 2015 relative à la facturation des antiviraux d'action directe (AAD) pour les patients pris en charge en ambulatoire dans des unités sanitaires en milieu pénitentiaire. https://www.legifrance.gouv.fr/circulaire/id/39572. 2021

[19] Adu PA, Rossi C, Binka M (2020). HCV reinfection rates after cure or spontaneous clearance among HIV-infected and uninfected men who have sex with men. Liver Int.

[20] HauteAutorité de Santé (2019).

[21] Thiara G, Sockalingam S. (2015). Psychiatric care of patients with hepatitis C: a clinical update. Psychiatr Times.

[22] Ehret MJ, Wang M. (2013). How to increase medication adherence: what works?. Ment Health Clin.

[23] Sockalingam S, Tseng A, Giguere P, Wong D. (2013). Psychiatric treatment considerations with direct acting antivirals in hepatitis C. BMC Gastroenterol.

[24] Miarons M, Sánchez-Ulayar A, Sempere G, Marín S, Castellví JM. (2019). New direct-acting antivirals for hepatitis C treatment and neuropsychiatric symptoms in psychiatric risk groups. Eur J Hosp Pharm.

[25] Rolland B, Bailly F, Cutarella C (2021). Hépatite C en milieu psychiatrique: un réservoir oublié?. L'Encéphale.

