# Supplementary material for: Impact of extending direct antiviral agents (DAA) availability in France: an observational cohort study (2015-2019) of data from French administrative healthcare databases (SNDS)
Source: Lancet Reg Health Eur. 2021 Dec 11;13:100281. doi: 10.1016/j.lanepe.2021.100281 (PMC8671622; doi:10.1016/j.lanepe.2021.100281)
Supplement: Supplementary file 1 [file mmc1.pdf]

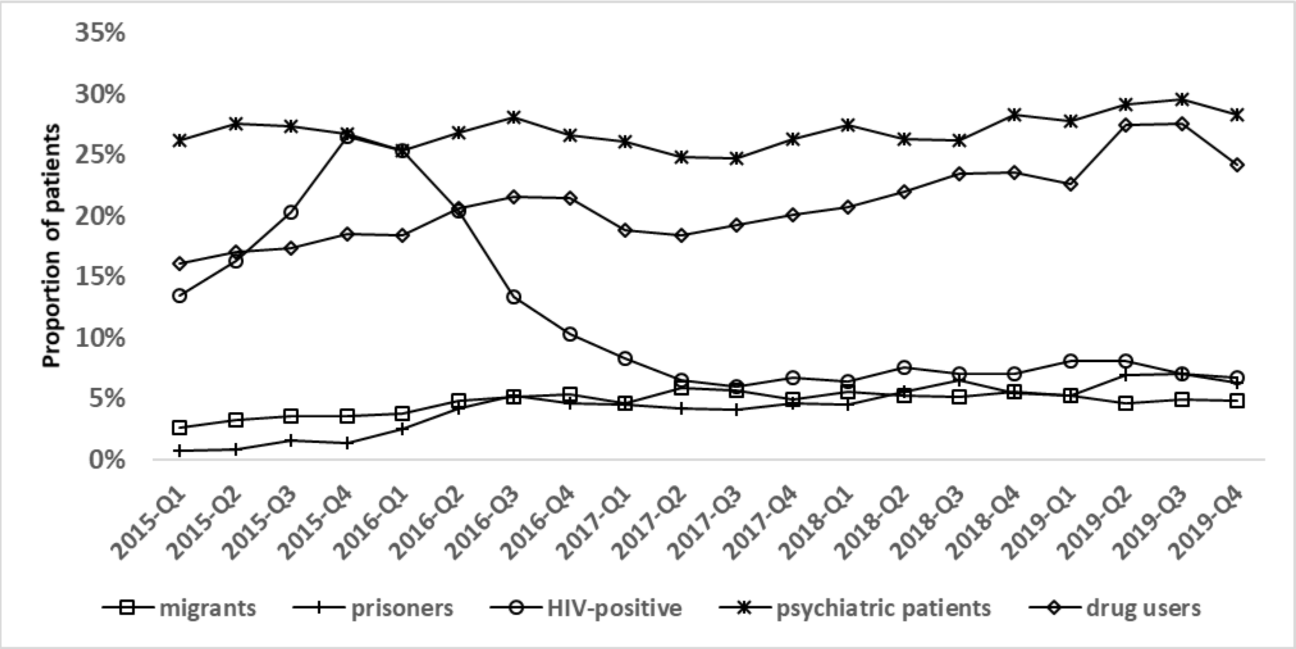

Supplementary Figure S1. Distribution of nonexclusive at-risk populations among the patients with treatment initiation, 2015-2019
