# Supplementary figures and images for: Impact of extending direct antiviral agents (DAA) availability in France: an observational cohort study (2015-2019) of data from French administrative healthcare databases (SNDS)
Source: Lancet Reg Health Eur. 2021 Dec 11;13:100281. doi: 10.1016/j.lanepe.2021.100281 (PMC8671622; doi:10.1016/j.lanepe.2021.100281)

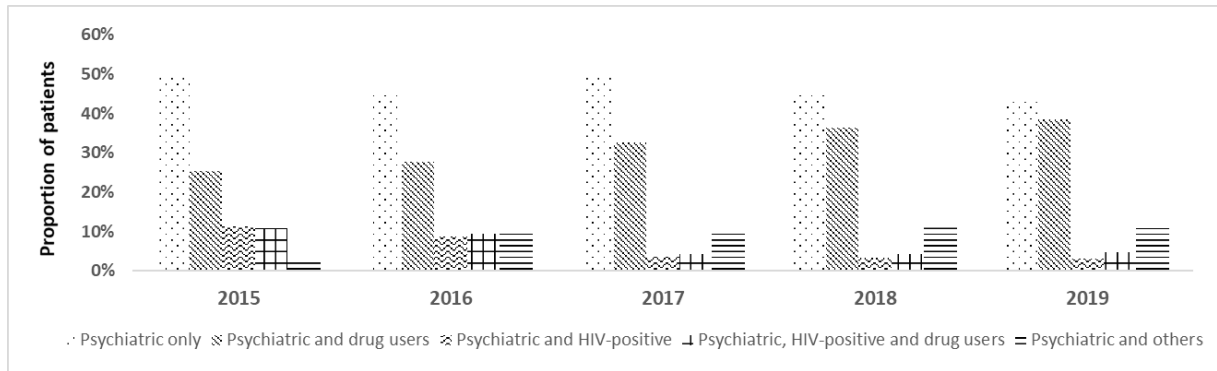

**Supplementary Figure S2. Distribution of the psychiatric subpopulation per year, 2015-2019**

Supplement: Supplementary file 2 [file mmc2.pdf]
