# Supplementary material for: Impact of extending direct antiviral agents (DAA) availability in France: an observational cohort study (2015-2019) of data from French administrative healthcare databases (SNDS)
Source: Lancet Reg Health Eur. 2021 Dec 11;13:100281. doi: 10.1016/j.lanepe.2021.100281 (PMC8671622; doi:10.1016/j.lanepe.2021.100281)
Supplement: Supplementary file 3 [file mmc3.pdf]

Supplementary Table S1. Algorithms and codes used to define the at-risk-population :

The "at-risk population" was identified through the following algorithms and according to the codes given in the following Table:

- Migrants: Individuals beneficiaries of the government run plan covering aliens with an irregular status (aide médicale d'état, AME);
- Prisoners: Individuals under the prisoners health insurance regime;
- HIV+ patients: Individuals diagnosed with HIV during a hospital stay or LTD for HIV or receiving an anti-HIV treatment;
- Psychiatric patients: Individuals diagnosed with a psychotic disorder, neurotic or mood disorders, addictive disorders or mental deficiency during a hospital stay and/or receiving antidepressants or LTD for these pathologies;
- Drug users (DUs): Individuals diagnosed with dependence/use of opioid drugs during a hospital stay and/or receiving opioid substitution therapy (OST) or beneficiaries of special insurance coverage for patients with chronic diseases.

Risk factor	**Algorithm**	**Diagnostic of hospitalization and/or LTD (ICD-10 codes)**	**Drug (Anatomical Chemical (ATC) classification system code)**	**Biological test (French Medical Common Procedure Coding System)**
HIV+ patients	Individuals diagnosed with HIV during a hospital stay or LTD for HIV or receiving an anti-HIV treatment	In MCO data (hospitalization of medicine, obstetric, surgery) : primary diagnosis*, related diagnosis**, significant associated diagnosis***<br>In RIM-P data (Collection of Medical Information in Psychiatry): primary diagnosis, significant associated diagnosis<br><br>B20 -B21: Human immunodeficiency virus [HIV] disease<br>F02.4: Dementia in human immunodeficiency virus [HIV] disease<br>Z20.6: Contact with and (suspected) exposure to human immunodeficiency virus [HIV]<br>Z21: Asymptomatic human immunodeficiency virus [HIV] infection status	J05AF01, J05AF02, J05AF03, J05AF04, J05AF05, J05AF06, J05AF07, J05AF09, J05AG01, J05AG03, J05AG04, J05AG05, J05AR01, J05AR02, J05AR03, J05AR04: Reverse transcriptase inhibitors<br>J05AE01, J05AE02, J05AE03, J05AE04, J05AE05, J05AE07, J05AE08, J05AE09, J05AE10, J05AR10: Protease inhibitors<br>J05AR06, J05AR08, J05AR09, J05AX07, J05AX08, J05AX09: Other HIV antivirals	0805: VIH: Genotypic resistance to antiretrovirals (Enzyme inhibitors)<br>0806: Genotypic resistance to antiretroviral drugs (Fusion inhibitors,...)<br>0388: HIV infection 1 and 2: Serodiagnosis detection<br>0389: HIV infection 1 and 2: Confirmation serodiagnosis 1 reaction<br>0390: IHIV infection 1 and 2: Confirmation serodiagnosis 2 or more reactions<br>0392: HIV infection 1 and 2: P24 antigen of HIV 1: research and titration

Risk factor	**Algorithm**	**Diagnostic of hospitalization and/or LTD (ICD-10 codes)**	**Drug (Anatomical Chemical (ATC) classification system code)**	**Biological test (French Medical Common Procedure Coding System)**
Psychiatric patients	Individuals diagnosed with a psychotic disorder, neurotic or mood disorders, addictive disorders or mental deficiency during a hospital stay and/or receiving antidepressants or LTD for these pathologies	In MCO data (hospitalization of medicine, obstetric, surgery) : primary diagnosis, related diagnosis, significant associated diagnosis<br>In RIM-P data (Collection of Medical Information in Psychiatry): primary diagnosis, significant associated diagnosis<br><br>F70-F79: Mental retardation<br>F10-F19: Mental and behavioral disorders due to use of psychoactive substances<br>F20-F29: Schizophrenia, schizotypal disorder and delusional disorders<br>F30-F39: Mood disorders [emotional]<br>F40-F48: Psychotic, stress-related and somatoform disorders<br>F80-F89: Psychological developmental disorders<br>F90- F98: Behavioral and emotional disorders usually appearing during childhood and adolescence<br>F04-F09: Organic mental disorders, including symptomatic disorders<br>F50 - F59: Behavioral syndromes associated with physiological disorders and physical conditions<br>F60-F69: Personality and behavior disorders in adults<br>F99: Mental disorder, unspecified	N05A Antipsychotics<br>N06A Antidepressants<br>N03AG02 Valpromides<br>N03AG01 Valproic acid (Depakote only)	N/A
Drug users (DUs)	Individuals diagnosed with dependence/use of opioid drugs during a hospital stay and/or receiving opioid substitution therapy (OST) or beneficiaries of special insurance coverage for patients with chronic diseases.	In MCO data (hospitalization of medicine, obstetric, surgery) : primary diagnosis, related diagnosis, significant associated diagnosis<br>In RIM-P data (Collection of Medical Information in Psychiatry): primary diagnosis, significant associated diagnosis<br>In SSR data (follow-up care and rehabilitation): all diagnosis<br><br>F11-F19: Mental and behavioral disorders due to use of psychoactive substances<br>T40: Intoxication by narcotics and psychodysleptics [hallucinogens]<br>Z715: Advice and monitoring for drug addiction and dependence<br>Z722: Drug use	N07BC01 Bueprenorphine<br>N07BC02 Methadone<br>N07BB04 Naltrexone<br>N07BC51 Buprenorphine in combination<br>V03AB15 Naloxone	N/A
Migrants	Individuals beneficiaries of the government run plan covering aliens with an irregular status (*aide médicale d'état*, AME)	N/A	N/A	N/A

Risk factor	**Algorithm**	**Diagnostic of hospitalization and/or LTD (ICD-10 codes)**	**Drug (Anatomical Chemical (ATC) classification system code)**	**Biological test (French Medical Common Procedure Coding System)**
Prisoners	Individuals under the prisoners health insurance regime	N/A	N/A	N/A

*Primary diagnosis: the health problem that motivated the patient's admission to the medical unit and established at discharge.
**Related diagnosis: report on the management of the patient in association with the primary diagnosis when this is not sufficient.
***Significant associated diagnosis: a condition, symptom or any other reason for healthcare use coexisting with the primary diagnosis, and constituting: an additional distinct health problem (another condition) or a complication of the primary condition or a complication of the treatment of the primary condition.
