# Supplementary material for: Impact of extending direct antiviral agents (DAA) availability in France: an observational cohort study (2015-2019) of data from French administrative healthcare databases (SNDS)
Source: Lancet Reg Health Eur. 2021 Dec 11;13:100281. doi: 10.1016/j.lanepe.2021.100281 (PMC8671622; doi:10.1016/j.lanepe.2021.100281)
Supplement: Supplementary file 4 [file mmc4.pdf]

Supplementary Table S2. Quarterly duration of DAA treatment in days, 2015-2019

Year	2015-Q1	2015-Q2	2015-Q3	2015-Q4	2016-Q1	2016-Q2	2016-Q3	2016-Q4	2017-Q1	2017-Q2	2017-Q3	2017-Q4	2018-Q1	2018-Q2	2018-Q3	2018-Q4	2019-Q1	2019-Q2	2019-Q3	2019-Q4
**n***	3,774	3,344	3,057	3,426	3,780	3,488	3,278	4,081	5,586	5,606	4,515	4,442	4,217	4,138	3,264	3,441	3,335	2,911	2,514	2,615
**Median**	84.0	84.0	84.0	84.0	83.0)	83.0	82.0	82.0	81.0	80.0	80.0	81.0	80.0	72.0	75.0	76.0	76.0	76.0	73.0	52.0
**Q1 - Q3**	[79.0-89.0]	[79.0 - 87.0]	[79.0-88.0]	[80.0 - 88.0]	[79.0 - 85.0]	[74.0-85.0]	[66.0-84.0]	[57.0-84.0]	[59.0-84.0]	[56.0-84.0]	[56.0-84.0]	[56.0-84.0]	[56.0-84.0]	[54.0-83.0]	[54.0-84.0]	[54.0-84.0]	[54.0-83.0]	[54.0-84.0]	[53.0-83.0]	[28.0-61.0]

** line of treatment number*
