# Supplementary material for: Impact of extending direct antiviral agents (DAA) availability in France: an observational cohort study (2015-2019) of data from French administrative healthcare databases (SNDS)
Source: Lancet Reg Health Eur. 2021 Dec 11;13:100281. doi: 10.1016/j.lanepe.2021.100281 (PMC8671622; doi:10.1016/j.lanepe.2021.100281)
Supplement: Supplementary file 5 [file mmc5.pdf]

Supplementary Table S3. Sociodemographic profile of the psychiatric population per year, 2015-2019

	Year	2015	2016	2017	2018	2019
**Age, years**	**N**	3628	3791	4958	3747	3000
	**Median**	54.0	53.0	53.0	53.0	53.0
	**Q1-Q3**	[49.0 - 60.0]	[48.0 - 59.0]	[47.0 - 59.0]	[46.0 - 59.0]	[46.0 - 59.0]
**Males**	**N** (%)	2381 (65.6)	2387 (63.0)	2942 (59.3)	2310 (61.6)	1965 (65.5)
