# Supplementary material for: Impact of extending direct antiviral agents (DAA) availability in France: an observational cohort study (2015-2019) of data from French administrative healthcare databases (SNDS)
Source: Lancet Reg Health Eur. 2021 Dec 11;13:100281. doi: 10.1016/j.lanepe.2021.100281 (PMC8671622; doi:10.1016/j.lanepe.2021.100281)
Supplement: Supplementary file 6 [file mmc6.pdf]

Supplementary Table S4. Characteristics of the retreated population, 2015-2019

		Total<br>N = 2,212
**Retreated patients***	**N (%)**	2,212 (3.7)
**Age, year**	**Median [Q1-Q3]**	55 [48-62]
**Males**	**N (%)**	1,557 (70.4)
**At-risk patients**	**N (%)**	1,163 (52.6)
**Psychiatric patients**	**N (%)**	634 (28.7)
**Drug users**	**N (%)**	112 (21.8)
**HIV-positive patients**	**N (%)**	514 (23.2)
**Migrants**	**N (%)**	121 (5.5)
**Prisoners**	**N (%)**	483 (5.1)
**Interval between the first and second course, months**		
**[3 - 6]**	**N (%)**	438 (19.8)
**[6 - 12]**	**N (%)**	704 (31.8)
**>12**	**N (%)**	1,070 (48.4)

*among incident treated patients (N = 71,466)
